# Relationship between Upper Extremity Lymphatic Drainage and Sentinel Lymph Nodes in Patients with Breast Cancer

**DOI:** 10.1155/2019/8637895

**Published:** 2019-04-01

**Authors:** Xiaokai Ma, Shishuai Wen, Baofeng Liu, Dumin Li, Xiaolong Wang, Xiaoli Kong, Tingting Ma, Liyu Jiang, Qifeng Yang

**Affiliations:** ^1^Department of Breast Surgery, Qilu Hospital, Shandong University, Jinan, Shandong 250012, China; ^2^Department of Oncological Surgery, The First Affiliated Hospital of Bengbu Medical College, Bengbu 233004, China; ^3^Department of Radiology, Qilu Hospital, Shandong University, Jinan, Shandong 250012, China; ^4^Department of Pathology Tissue Bank, Qilu Hospital, Shandong University, Jinan, Shandong 250012, China

## Abstract

**Purpose:**

The purpose of this study was to identify the relationship between upper extremity lymphatics and sentinel lymph nodes (SLNs) in breast cancer patients.

**Methods:**

Forty-four patients who underwent axillary reverse mapping (ARM) during axillary lymph node dissection (ALND) with SNL biopsy (SLNB) between February 2017 and October 2017 were investigated. ARM was performed using indocyanine green (ICG) to locate the upper extremity lymphatics; methylene blue dye was injected intradermally for SLN mapping.

**Results:**

ARM nodes were found in the ALND fields of all examined patients. The rate of identification of upper extremity lymphatics within the SLNB field was 65.9% (29 of 44). The ARM nodes were involved in metastases arising from primary breast tumors in 7 of the patients (15.9%), while no metastases were detected in pathologic axillary lymph node-negative patients. Lymphatics from the upper extremity drained into the SLNs in 5 of the 44 patients (11.4%); their ARM-detected nodes were found to be in close proximity to the SLNs.

**Conclusions:**

The ARM nodes and SLNs are closely related and share lymphatic drainage routes. The ARM procedure using fluorescence imaging is both feasible and, in patients who are SLN negative, oncologically safe. ARM using ICG is therefore effective for identifying and preserving upper extremity lymphatics, and SLNB combined with ARM appears to be a promising surgical refinement for preventing upper extremity lymphoedema.

**Clinical Trial Registration:**

This trial is registered with ClinicalTrial.gov: NCT02651142.

## 1. Introduction

Approximately 232,340 new cases of invasive breast cancer and 39,620 breast cancer-related deaths were expected to occur among women in the United States in 2013 [1]. Patients with primary clinical node-positive breast cancer often require modified radical mastectomy, and axillary lymph node dissection (ALND) is a gold-standard surgical approach. However, ALND is associated with substantial morbidity, as upper extremity lymphedema occurs in 7–77% of patients [2–7] and leads to arm/shoulder dysfunction, paresthesia, and discomfort [8].

Sentinel lymph node (SLN) biopsy (SLNB) has gradually replaced ALND in clinical lymph node-negative patients [9–12]. Although SLNB has significantly reduced the incidence rate of complications compared with ALND, some patients still experience chronic arm pain or even lymphoedema. Clinical trials have shown that upper extremity lymphoedema occurs in 2–7% of patients who undergo only SLNB [13–15].

In 2007, axillary reverse mapping (ARM) was developed as a tool to distinguish lymph nodes in the upper extremity from those in the axillary region belonging to the breast lymphatic drainage system [16, 17]. The fundamental concept of ARM is based on the hypothesis that the upper limb and the breast each have their own independent lymphatic drainage systems through the axilla. If postoperative upper extremity lymphoedema is due to the disruption of upper limb lymphatics, then identifying them via ARM and preserving them could potentially reduce the occurrence of such lymphoedema following ALND or SLNB [18].

In the present study, we investigated the relationship between upper extremity lymphatics and SLNs in patients with breast cancer to determine if the lymphatic drainage system of the upper extremity is, in fact, completely independent of that of the breast. To clarify the anatomy, all the patients first underwent SLNB and ARM, after which level 3 axillary dissections were performed. We aimed to identify the feasibility and oncologic safety of the ARM technique as well as the possible causes of lymphoedema in the upper extremities following SLNB.

## 2. Patients and Methods

### 2.1. Patients

This study included 44 women with stage I–III breast cancer who underwent ARM during ALND with SLNB at our institution between February and October, 2017. Their ages ranged from 29 to 76 years with a median of 51 years. Three patients underwent neoadjuvant chemotherapy, although none were given hormone therapy before surgery. All patients were informed of the aims of the research as well as the potential effects and risks of the procedures, and all signed a consent form approved by the Medical Ethics Committees of Qilu Hospital of Shandong University.

### 2.2. SLNB

SLNB was performed with methylene blue (10 mg/mL), a 1 mL dose of which was injected intradermally into the periareolar skin as well as the skin overlying the tumor. SLNs were detected following the migration of the blue dye through the lymphatics.

### 2.3. ARM and ALND Procedures

A 1 mL dose of indocyanine green (ICG; 0.5 mg/mL) was injected subcutaneously into the upper inner arm 1 hour before surgery. We performed a 2-minute surface massage at the injection site to promote upper limb lymph flow. Approximately 10 minutes later, fluorescent ARM lymphatic drainage pathways were detected from the point of injection to the axillary nodes, after which total mastectomy and ALND were performed. During ALND, an invisible near-infrared fluorescence real-time dynamic imaging system was used to identify the ARM nodes and/or lymphatics. When ICG flows through the ARM nodes and/or lymphatics, the fluorescent spots and streams are observable in real time on the monitor. We recorded the nodes detected by ARM and measured the distance between them and the SLNs. ALND was subsequently performed to remove the ARM-detected nodes, and both the SLNs and ARM-detected nodes separately underwent pathological examination.

### 2.4. Statistical Analysis

All clinical data were analyzed using SPSS 22.0 (IBM Corp., Armonk, NY). The chi-square test was used to analyze the correlation between ARM-detected and axillary lymph node metastases. All *p*-values were 2-tailed, and* p* < 0.05 was considered statistically significant.

## 3. Results

### 3.1. Patients

A total of 44 patients who successfully underwent simultaneous ARM, SLNB, and ALND were included in this study. Of these patients, 40 had invasive ductal carcinomas, 3 had invasive ductal carcinoma and high-risk intraductal carcinoma, and 1 had metaplastic carcinoma. Of the 44 invasive carcinomas, 24 (54.5%) were T1, 19 (43.2%) were T2, and 1 (2.3%) was T3. In terms of the histologic grade, 4 (9.1%) were G1, 21 (47.7%) were G2, and 19 (43.2%) were G3.

### 3.2. SLNB

The mean number of SLNs removed was 1.8 (range, 1–4). Among all 44 patients, the SLNs were histologically positive in 21 (47.7%); the numbers of positive SLNs were 4 in 1 patient, 2 in 1 patient, and 1 in 19 patients.

### 3.3. ARM

Using ICG, 182 fluorescent ARM nodes were identified and removed from the 44 patients. The mean number of removed ARM-detected nodes was 4.1 (range, 1–12); the rate of positive nodes was 9.3% (17 of 182). Among all 44 patients, the ARM-detected nodes were histologically positive in 7 (15.9%). Moreover, 33.3% (7 of 21) and 0% (0 of 23) of the ARM-detected nodes in pathologic SLN-positive and pathologic SLN-negative patients, respectively, were metastatic (Table 1).

### 3.4. The Relationship between ARM-Detected Nodes and SLNs

**Figure 1:**
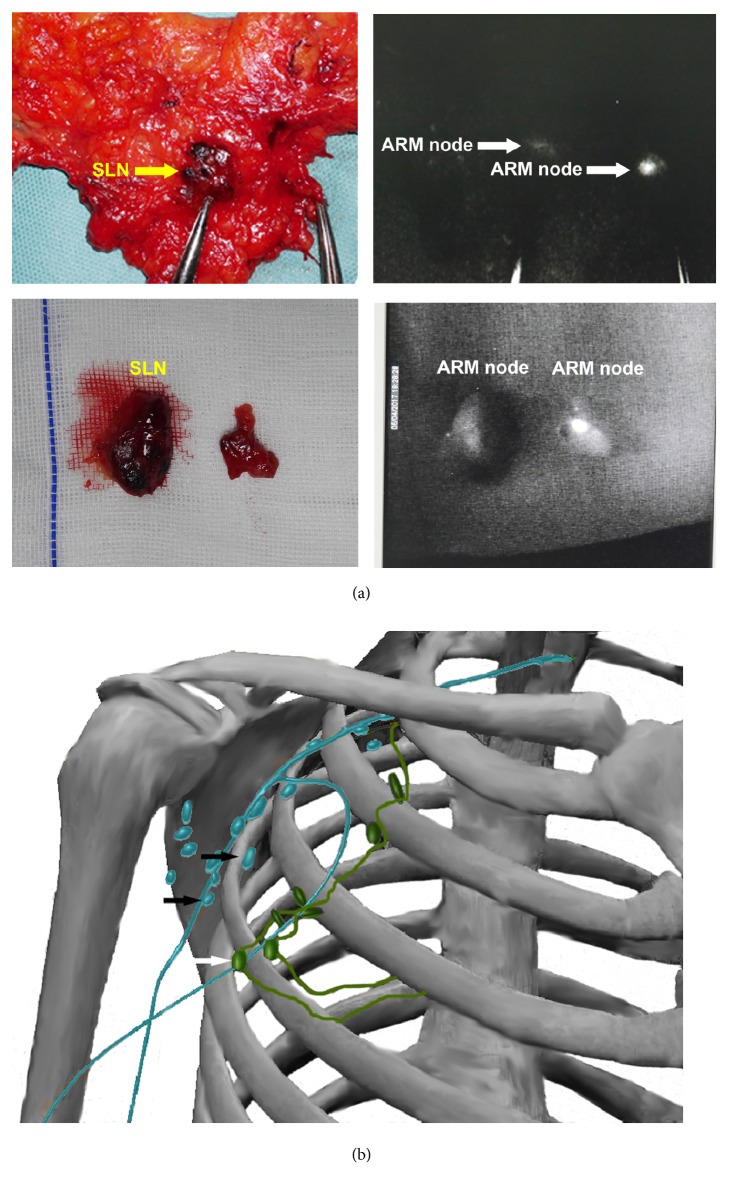
Equivalent findings on sentinel lymph node (SLN) biopsy ((a), left panels) and axillary reverse mapping (ARM) ((a), right panels). A diagram of the lymphatics is shown in (b); the blue-colored lymphatic duct is that of the upper limb. Yellow arrows indicate biopsied SLNs, while white arrows indicate ARM-detected nodes.

We found that the lymphatics of the upper extremity and breast were converged through the same node in some of the patients who successfully underwent SLNB and ARM. The SLN was the same as the ARM-detected node in 5 patients (11.4%) (Figure 1). The ARM-detected node was the same as that located in the next SLN station in 4 patients (9.1%; designated posterior SLN). In the remaining 35 patients, the nearest distances (D, cm) between the SLN and ARM nodes are shown in Table 2. Additionally, ARM detected level 3 nodes in 5 patients, level 2 nodes in 3 patients, and nodes along the fat that lies behind the clavipectoral fascia and is superoanterior to the axillary vein (designated tongue-shaped fat) in 7 patients.

### 3.5. Patients with Metastatic ARM Nodes

Of the 7 patients with ARM-detected metastatic nodes, 5 were T1, 2 were T2, 2 were pN1, 3 were pN2, and 2 were pN3. Among these 7 patients, the metastatic ARM-detected node was also the SLN in 1 patient, while the nearest distance between the metastatic ARM-detected node and SLN in the remaining 6 patients was >2 cm. Six of the 7 patients with metastatic ARM-detected nodes had luminal B type disease while 1 had luminal A type. Of the 37 patients without metastatic ARM-detected nodes, 16 had luminal B type, 9 had luminal A type, 10 had triple-negative disease, and 2 were HER-2 positive (Table 3).

## 4. Discussion

Although postoperative lymphedema occurs in a minority of patients, its development markedly diminishes the quality of life [19]. SLNB, which can help avoid postoperative upper extremity lymphoedema, has gradually replaced ALND as a minimally invasive procedure in clinically node-negative patients. However, 6.9% of the patients investigated in the American College of Surgeons Oncology Group Z0010 trial demonstrated proximal upper extremity lymphoedema (defined as a change in baseline arm circumference of >2 cm) following SLNB [13]. Because different patients may have distinct lymphatics and different numbers of SLNs [20, 21], some of these nodes may be missed during SLNB. Previous studies have demonstrated a positive correlation between the number of SLNs and false-negative rates [22, 23]. Hence, a greater number of non-SLNs may be removed to reduce the false-negative rates during SLNB.

Upper extremity lymphoedema may be caused by the resection of the upper-limb lymph nodes and lymphatics. The greater the number of resected non-SLNs, the greater the probability of occurrence of upper-limb edema. In our previous study [24], we identified all the true SLNs, para-SLNs, and post-SLNs by following the lymphatic drainage ducts. After precisely locating the lymphatic channels, all the lymph nodes that first received lymphatic drainage were designated true SLNs, following which we precisely distinguished the true SLNs, para-SLNs, and post-SLNs. This prompted our curiosity regarding the relationship between the true SLNs and para-SLNs or post-SLNs; i.e., whether the para-SLNs and post-SLNs were drained from the upper limbs. Therefore, we performed ARM, which was originally used to study the relationship between the lymphatics of the upper extremities and breasts, in patients undergoing SLNB.

The purpose of the ARM procedure is to preserve the upper-limb lymph nodes and lymphatics, which in turn can help reduce the occurrence of upper extremity lymphoedema. Previous studies have confirmed the anatomical position of the ARM-detected nodes, which lie in the upper outer quadrant of the axilla just caudal to the axillary vein and lateral to the ascending lateral thoracic vein that ends in the axillary vein [18]. Tummel et al. [25] performed 685 ARM procedures during SLNB and/or ALND and found that the crossover rates were 3.8% for SLNB and 5.6% for ALND. Objective lymphoedema was observed in 0.8% of patients undergoing SLNBs and 6.5% of those undergoing ALNDs. In relation to accepted standards, the rates of upper extremity lymphoedema were significantly reduced when ARM was used. Furthermore, it was previously reported that ARM during SLNB can also identify patients who are susceptible to developing lymphoedema [26].

Crossover refers to lymph nodes from the upper limb and breast leading to the axilla through a common pathway; such nodes are referred to as SLN-ARM nodes in previous studies [18, 27]. We found some anatomical variations between patients in our study. The SLNs were the same as the ARM-detected nodes in 3 patients, and recurrent nodes in the upper extremities of these patients would inevitably be removed during SLNB; hence, these patients were more likely to have upper-limb lymphoedema. The ARM-detected node was the same as that located in the next station in 4 patients (designated post-SLN). Post-SLNs were also dyed in blue and could be easily mistaken for SLNs and removed, thus rendering these patients susceptible to developing upper-limb lymphoedema. To clarify the anatomical positioning of the ARM-detected nodes, Ikeda et al. [28] divided the axilla into 5 areas; the ARM nodes were mostly located between the axillary vein and the second intercostobrachial nerve close to the anterior edge of the latissimus dorsi (field A). In our study, the ARM-detected nodes were mostly located in the upper-outer quadrant of the axilla, below the second intercostobrachial nerve, or on the lateral side of the thoracodorsal nerve. The distances between the ARM-detected nodes and SLNs were different; those within a range of 2 cm of the SLNs were considered to be within the “SLNB field”; we found that the ARM-detected nodes in most patients (29 of 44, or 65.9%) were located in this SLNB field. Owing to the short distance and convergence between the ARM-detected nodes and SLNs, the former were easily excised simultaneously with the latter during SLNB. This may explain the occurrence of upper-extremity lymphoedema. Additionally, ARM-detected nodes were present in level 3 in 2 of our patients, which may also explain why level 3 node excision can cause lymphedema.

The feasibility of ARM and its oncologic safety remain important concerns. Nos et al. [29] achieved a node detection rate via ARM of 100%; 31% of the ARM-detected nodes were positive and 9% were in zone D. They also found that patients with pN1 disease had fewer positive ARM-detected nodes than those with pN2 or pN3 disease [30]. Using the split mapping technique for the upper limb and breast, Han et al. [31] obtained results that were similar to those of Tummel et al., which demonstrated that the ARM procedure was oncologically safe [25]. The node identification rate using ARM was 95.7% in our study (44 of 46); aside from 1 metastatic ARM node being the SLN itself, the remainder were located in the ALND field but not the SLNB field. The remaining patients with nodes at distances <3 cm and within the SLNB field had no metastatic ARM-detected nodes. These findings indicate that it is oncologically safe to retain ARM-detected lymph nodes during SLNB in patients with early breast cancer. However, as 15.9% of the ARM-detected nodes in our study were metastatic, the risk factors for potentially metastatic nodes detected using ARM ought to be identified. The small number of metastatic ARM-detected nodes in our study precluded the use of statistical analyses in this regard; however, it was apparent that patients with luminal B-type disease had a greater proportion of metastatic ARM-detected nodes than did patients with other types of breast cancer. Moreover, patients with metastatic ARM-detected nodes had 7.42 metastatic axillary lymph nodes on average, compared to 2.24 in those without metastatic ARM-detected nodes. However, no metastatic ARM-detected nodes were found in pathologic axillary and/or SLN-negative patients. Therefore, ARM-detected node preservation may be risky in patients with extensive metastases but appears to be safe for node-negative patients who meet the criteria for SLNB.

In a study of lymphatic drainage in the upper limb and mammary regions of 7 stillborn fetuses, Guilherme et al. [32] discovered numerous communicating lymphatic vessels between the anterior and lateral axillary groups. The upper limb drained to the lateral axillary group in 8 of their 14 specimens (57.14%); however, the upper limb lymphatics in 5 of 14 specimens (35.71%) and those in the mammary regions in all fetuses drained into the anterior axillary group. Our present findings that the lymphatic drainage of ARM-detected nodes and SLNs are closely related were consistent with those of Guilherme et al.; the upper extremity lymphatics directly drained into the SLNs in 11.4% of our patients. Furthermore, 25.9% of our pathologic ALN-positive patients had histologically positive ARM-detected nodes. These findings indicate that the ARM-detected nodes were also involved in breast cancer metastasis and that preserved ARM-detected nodes may therefore harbor malignant cells.

In terms of choosing the appropriate tracer for the ARM procedure, both radionuclides and blue dyes have been used. Radionuclides such as technetium 99 may result in unwanted radiation exposure and is not available in some hospitals. Blue dyes such as methylene blue may cause lifelong skin markings. Fluorescence imaging for detecting upper extremity lymphatic drainage was first used for the ARM procedure by Noguchi et al. [33, 34]. We used ICG for the ARM procedure; no allergic reactions occurred and the skin coloration on the upper inner limb only lasted between 1 week and 2 months before disappearing. ARM using fluorescence imaging can effectively identify the upper limb reflux lymph nodes to improve their recognition and, thus, their preservation even though they are not necessarily dyed in blue. This improves the potential of reducing upper limb lymphoedema in patients undergoing SLNB.

## 5. Conclusions

The ARM procedure improves the precision of SLNB and is also feasible and oncologically safe. Because of the small number of patients in our study along with the lack of follow-up information, additional investigations that include multicenter collaborative studies ought to be performed to ensure higher data reliability.

## Figures and Tables

**Table 1:** Metastasis rates in ARM-detected nodes vs. axillary lymph nodes.

		ARM node metastasis		*χ* ^2^	*p*-value
		Positive	Negative		
ALN metastasis	+	7	20	3.5	0.062
	-	0	17		
SLN metastasis	+	7	14	6.8	0.009
	-	0	23		

ARM, axillary reverse mapping; ALN, axillary lymph node; SLN, sentinel lymph node.

**Table 2:** Surgical findings.

	SLNB+ARM+ALND (n=44)
Blue SLN	80
Mean number of blue SLNs	1.8
Blue SLN positive for malignancy	21

Fluorescent ARM nodes	182
Mean number of ARM nodes	4.1
ARM nodes histologically positive	17
ARM-SLN	5
ARM-posterior SLN	4

Distance between ARM nodes and SLN	
D1 (0 cm)	3
D2 (>0 and ≤1 cm)	7
D3 (>1 and ≤2 cm)	10
D4 (>2 and ≤3 cm)	7
D5 (>3 and ≤4 cm)	3
D6 (>4 and ≤5 cm)	3
D7 (>5 cm)	2

ARM, axillary reverse mapping, SLN, sentinel lymph node; SLNB, sentinel lymph node biopsy; ALND, axillary lymph node dissection; D, closest distance between the SLNs and ARM-detected nodes.

**Table 3:** Characteristics of patients with and without positive metastatic ARM nodes.

	Metastatic ARM nodes (n=7)	Non-metastatic ARM nodes (n=37)
Median age (range), years	51 (29–74)	58 (44–76)

T		
≤2 cm	5	20
2–5 cm	2	16
≥5 cm	0	1

N		
pN0	0	19
pN1	2	11
pN2	3	4
pN3	2	3

Luminal type		
A	1	9
B	6	16
HER2-positive	0	2
Triple-negative	0	10

ARM, axillary reverse mapping

## Data Availability

The data used to support the findings of this study are available from the corresponding author upon request.
